# Semantics in High-Dimensional Space

**DOI:** 10.3389/frai.2021.698809

**Published:** 2021-08-31

**Authors:** Jussi Karlgren, Pentti Kanerva

**Affiliations:** ^1^ Gavagai, Stockholm, Sweden; ^2^ Redwood Center for Theoretical Neuroscience, University of California at Berkeley, Berkeley, CA, United States

**Keywords:** vector-space models, semantic spaces, high-dimensional computing, distributional semantics, word embeddings

## Abstract

Geometric models are used for modelling meaning in various semantic-space models. They are seductive in their simplicity and their imaginative qualities, and for that reason, their metaphorical power risks leading our intuitions astray: human intuition works well in a three-dimensional world but is overwhelmed by higher dimensionalities. This note is intended to warn about some practical pitfalls of using high-dimensional geometric representation as a knowledge representation and a memory model—challenges that can be met by informed design of the representation and its application.

## Introduction

Vector representations are used for several computationally important tasks that involve knowledge representation. This is for good reason: vectors are a useful representation for cases that involve aggregating information to learn about things we observe. Continuous vector representations are convenient for modelling knowledge that is unsuitable for fixed and definitional representations, or knowledge that has not yet settled into something we are certain about, especially in situations where new data keep arriving to motivate us to adjust what we know. Vector representations allow us to compile large amounts of data into a convenient, relatively compact, emergent representation.

Vectors also provide a convenient interface to downstream computation using various flavours of machine learning: a set of vectors aggregated from observed data can be fed into any of several effective machine-learning models.

Vectors need to be processed somehow for us to understand what we are working with and how the things we observe relate to each other. A vector space gives us a way to understand the items represented in it and how they relate to each other. Spatial models invite us to view distance and nearness as relevant: spatial reasoning comes with a convenient and well-understood geometric processing model and a useful palette of metaphors with which we can talk about what we learn.

## The Promise of Continuous Representations

Discrete symbolic models have obvious drawbacks for processing human language. Linguistic expression can be polysemous, indeterminate, vague, unspecific, and non-exclusive: any concept or notion can be referred to in more or less equivalent ways, the ways in which those ways differ is difficult to formalise, and most every utterance and constituent of an utterance can be recruited or coerced to be relevant for situations that are different from the most prototypical ones it can be expected to appear in. By implementing a continuous rather than a discrete representation, many of those challenges appear to be less pressing.

Observable functional similarities between linguistic items, both constructional and lexical, are sometimes discrete and sometimes gradual. Gradual similarities fit well in a continuous representation (Bengio et al., 2003, e.g., argue this point well). The similarity between the utterances given in Example (1) demonstrates how the pairwise similarity between some of the constituents helps establish that the utterances are similar in meaning in certain ways, and a system to learn e.g., constructional patterns or entailments would be well served to make use of the similarities. Experience with processing horses will help when a donkey turns up for the first time.

1) a. The horses need their oats and hay.
b. Everyone who owns a donkey has given it hay and carrots.

This is where a vector-space model can come in handy. A vector representation allows us to model similarity between items on a continuous scale, and with an increased dimensionality several different similarities between items can be accommodated without blocking each other. This provides a basis for an automatic generalisation from terms to concepts, which is one of the fundamental motivators for a distributed or connectionist approach (Hinton et al., 1986).

Modelling linguistic items by assigning a vector to each, allows us to use a geometric metaphor for meaning. The clear-cut formulation by Schütze (1993), *“vector similarity is the only information present in Word Space: semantically related words are close, unrelated words are distant,”* indicates how distance in a geometric space can be interpreted as a measure of semantic relatedness. The vector-space representations come with operations to combine and compare constituents in a well-understood and computationally habitable algebraic framework. Probably the first vector-space formulation is by H.P. Luhn (1953): *“If we consider a concept as being a field in a multi-dimensional array, we may then visualize a topic as being located in that space which is common to all the concept fields stated. It may further be visualized that related topics are located more or less adjacent to each other depending on the degree of similarity and that this is so because they agree in some of the identifying terms and therefore share some of the concept fields.”* Vector-space models became and have remained the semantic basis of information-retrieval system (Salton et al., 1975; Dubin, 2004), while computational linguistics for a long time favoured symbolic representations. Semantic-space models were also built by psycholinguists and behaviourists, based on meaningful and preselected dimensions, and hand-encoded (Osgood et al., 1957, most notably), to be validated in psychological experiments. In such settings, test subjects scored items of interest numerically along a small number of postulated basic semantic dimensions such as “small vs. large” and “rough vs. polished.” In the 1990’s, statistical language-processing methods and connectionist memory models converged with semantic spaces to formulate models where the makeup of the space emerges from the data instead of being preselected (Deerwester et al., 1990; Lund and Burgess, 1996; Kanerva et al., 2000). These models form the basis for today’s word-embedding models of various kinds.

## Similarity and Distance

The notion that some items of interest are *close* to each other and others are *far* from each other, and that in the *neighbourhood* of something interesting we will expect other interesting things to be found, is catchy and easy to explain. Examples (2-a) and (2-b) demonstrate how natural it seems to us to use a geometric metaphor for semantic similarity. The notion of semantic distance is frequently used and mostly left unexplained as a primitive but observable effect in psycholinguistics and related research fields (Rips et al., 1973).

2) a. Is coffee close to tea in meaning?
b. Is coffee closer to tea than to wine?

On another level, vector-space models are intuitive since they conform somewhat to our current understanding of how our brains work. All of that is only partially true, but it contributes to our understanding of the utility of vector-based models.

On a third level, the notion of a continuous rather than a discrete representation appears to capture both the gradual learning of new relations and the observable fuzziness of human language, and thus to handle the occasional vagueness, polysemy, or unspecificity that linguistic expression exhibits. Modelling gradual learning with continuous representations is appropriate, but other characteristics of language are not always as well suited as they might seem to be at first glance. While distance can be used to model uncertainty, specifically in a learning situation, and might be useful for modelling polysemy, uncertainty^1^ is different from vagueness and unspecificity. Neither vagueness nor unspecificity [cf. sentences given in Examples (3-a) and (3-b)] are well modelled by point-wise distance measures: a single continuous representation can not be a cure-all for modelling the complexities of human language.

3) a. People who like this sort of thing will find this the sort of thing they like.
 b. The vehicle passed the object at a lower than average speed.

Intuitiveness is not always a good thing. The planetary model for understanding atoms and molecules, for example, leads into the wrong conclusion about the nature of electrons. We do not quite have intuitions to understand what high-dimensional space looks like and what distances mean. If we are in a 128-dimensional, 1,000-dimensional, or even 10-dimensional space, the natural sense of space, direction, or distance we have acquired poking around over our lifetime on the 2-dimensional surface of a 3-dimensional sphere do not quite cut it and risk leading us astray. This paper is intended to warn of some attendant methodological pitfalls.

## An Average is a Lossy Compression

Data in a vector space is mostly represented as *points*. In a learning scenario, as we obtain more and more observations of some item of interest in a noisy stream of sampled data, it does make sense to take the average or *centroid* of those observations to represent the item as a point in some way, possibly weighting or normalising the observations as we go. We move the point around to represent the item in the vector space, based on new information as it accrues. This is intuitively very attractive: the aggregation of observations seems to generalise nicely into a concept. But an average is a lossy compression of the original data set: is there reason to assume that a centroid is a meaningful representation?

If we have a number of observations from data and take their centroid to be the generalisation over them, we should make sure that this is a reasonable thing to do. If the notion we are tracking is complex or if we wish to combine several items into a compositional whole, an average might not make sense anymore.

For instance, if we are interested in hot beverages, collecting different observations of “*coffee*” to understand how it is used, is a good idea. We can also collect observations of several terms we know it might be related to such as “*cocoa*,” “*latte*,” “*espresso*,” “*tea*,” “*rooibos*,” and “*earl grey*” and join them to get a general hot beverage concept. This would seem to be sensible. The centroid of those terms is likely to hold other interesting neighbours such as “*macchiato*” and “*oolong*”. If we then would like to think about the notion “*fika*” we might want to consider various terms for drinking and imbibing, cups and mugs, cinnamon buns and ginger snaps, and so forth. In a geometric model, averaging out all these different notions is probably a bad idea (depending a little on how the terms and their context are collected from observed language): the “average” of the action of drinking, what is being drunk, and liquid containers is not a well-defined semantic notion, not even in a semantic space. Yet, the practice of folding diverse concepts into a centroid is quite common in semantic models, such as when a sentiment-analysis model is built from a set of diverse seed terms: the vector space is assumed to provide some sort of automatic generalisation of the items of interest, where it in fact is a collection of observations. A vector space adds nothing to the information it is built from.

Adding together e.g., all colour words into a centroid, all names of months into a centroid, or a set of expletives and lewd terms into a centroid does not yield a representation of colourfulness, of months, or of profanity.

This becomes especially true in the light of the variety of ways a semantic space is populated. Context models are sensitive to definition of what context is and what items are. The items under consideration can be words, multi-word terms, or constructions in the case of a lexical semantic space, they can be observed user action on various levels of granularity, they can be contextual data or various kinds of measurements. How the vectors that represent them are accrued also varies, for words ranging from close to broad contexts, which in various ways determines whether a set of items is suited for representation by an average vector (Sahlgren, 2006).

In every case a centroid is taken as a representation of several observations, this should be done with an awareness that items cannot typically be averaged to get something sensible but should be done based on a hypothesis that is motivated from both the type of item under consideration and the type of data used to aggregate the vector. A vector sum does not in itself provide a generalisation of the component features. It only provides a representation of their combination. Representation of concepts by *distributions* of points in a high-dimensional space rather than by an average of several points is a possible avenue of investigation that would introduce a probabilistic interpretation of overlaps of such distributions.

## The Emptiness of the “Polar Cap”

In whatever dimensionality we operate, we tend to be mostly interested in the *hypersphere* or *unit sphere* (the sphere centred on the origin and with a radius of 1). This is because we typically use the cosine as a similarity measure, which normalises vector length and compares angles between them. The points of interest in the high-dimensional space are scaled to fall on the surface of the hypersphere, and we are looking for structure in high-dimensional space as it is projected on to that surface. Now, as we move into higher and higher dimensionality, the hypersphere contains less and less of the volume of the enclosing hypercube (the cube centered on the origin with sides of length 2). An increasing majority of the points in a hypercube is in its corners and thus lies far from the surface of the hypersphere, and any projected structure in the original space that depends on the differences in distance from the origin is lost. The structures we pursue in the vector space are only partially shadowed onto the wall of the hypersphere cave.

**FIGURE 1:**
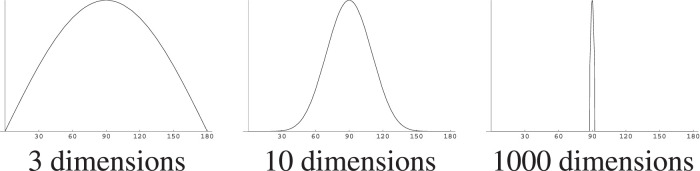
The distribution of angles between two randomly chosen points on spheres of various dimensionalities [Figure by Anders Holst, from Karlgren et al. (2008)].

This can be illustrated by first taking a hypersphere in 2-D: a circle. Pick a point—call it twelve o’clock—and then pick another point at random and record the angle between the vectors to those points. Those randomly picked angles are distributed uniformly between 0° and 180°. Now, in 3-D, where the hypersphere is what we usually think of as a sphere, again pick a point—call it the North Pole—and then pick random points on the surface of the sphere, again recording the angles. There are many more points on the equator, at 90° from the pole, than on the tropical or polar circles, and it is much more likely that a point picked at random is at a low latitude. The angles will be distributed along a bell-shaped curve. The pointedness of that bell curve continues to become more pronounced as dimensionality grows, as shown in Figure 1 which shows the probability distribution of the angle between two randomly chosen points in 3-, 10-, and 1,000-dimensional spaces.

For more mathematical background, see e.g., Kanerva (1988); Widdows (2004); Widdows and Cohen (2014).

This is important to understand for practical purposes because of how we relate *interestingness* to distance and prevalence: from any point of view the interesting things are close by and relatively few. We are used to the idea that if *A* is close to *B* and *A* also is close to *C*, then *B* cannot be very far from *C* or in other words if items *A* and *B* are at distance *d*_*a*,*b*_ and items *A* and *C* are at distance *d*_*a*,*c*_ then the distance *d*_*b*,*c*_ between items *B* and *C* is bounded by *d*_*a*,*b*_ + *d*_*a*,*c*_. The challenge is to understand why if distances *d*_*a*,*b*_ and *d*_*a*,*c*_ both are low enough to be interesting, why *d*_*b*,*c*_ might not need to be.

Since the distribution of angles is highly concentrated around 90°, points even slightly closer are likely to be interesting. If a point is at say 70° or even 80° from the North Pole in high dimensions this is very rare and something we should make note of. The *horizon of interest* extends further, as it were, as we move into higher dimensionalities. If *A* and *B* are at e.g., 70° from each other, and *A* and *C* likewise are at 70° from each other, this tells us next to nothing about how close *B* and *C* are. Interestingness is not transitive [a similar argument is given by Beyer et al. (1999)].

**FIGURE 2:**
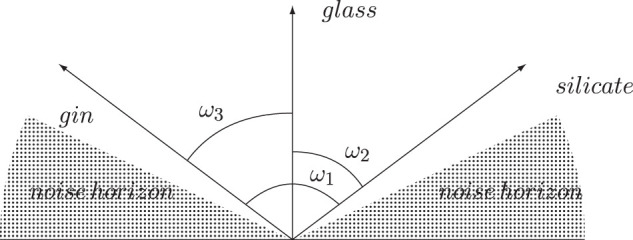
Interestingness is not always transitive.

This has as a consequence that the region of interesting and notably low distances between points grows while the noise corridor tightens up. In Figure 2 the distance *d*(*glass*, *gin*) =  cos(*ω*_3_) is about the same as the distance *d*(*glass*, *silicate*) =  cos(*ω*_2_) and while the angle between them is large, this is still an interesting distance compared to the noise at higher levels. However, the distance *d*(*gin*, *silicate*) =  cos(*ω*_1_) is too large to be notable and descends beneath the semantic horizon. This example from two dimensions becomes even more noticeable as the number of dimensions grows.

## Local Neighbourhoods and Global Structures

Most relations of interest in semantic spaces are local. Mostly, it is the neighbourhood we want more information about, not the global structure of the space. A semantic space typically knows of many things and most of them occur and are observed every now and then without connection to each other. Most of them have no definable relation at all and fit comfortably in a space of sufficiently many dimensions. As distances go, “close” is interesting and “distant” is not.

It is in the nature of human understanding of the world that relations between one thing and another thing emerge and grow to be interesting as events unfold, but most items of interest will remain uninteresting with respect to each other. This has the side effect that global structures based on semantic distance alone are likely not to be useful.

The above points call into question the entire notion of global semantic structure. There seems to be little reason to assume that the structure in the semantic space on a global scale holds any meaning.

We conclude that

• semantic spaces are interesting in view of their local structure and neighbourhood, not the global structure, and that
• any operation on a semantic space and any manipulation or transformation of a semantic space that would take purchase in its global structure is quite unlikely to be fruitful and very probably wasteful in terms of computation.

The idea that different semantic spaces could be translated to each other, if trained on similar data, is interesting and intuitively attractive. Some recent experiments have shown that it is possible to align a semantic space trained on one language with a semantic space trained on a comparable text collection in another, and this would seem to indicate that some such structure could be found (Alvarez-Melis and Jaakkola, 2018). For instance, if a lexical model or a word space is trained on text in one language and then another model is trained on text in another language, they might be translatable to each other through a distance-preserving transformation: after all, it is highly likely that the respective terms for “*boat*,” and “*Boot*” will have terms for “*ship*” or “*Schiff*” at similar distances and terms for “*spruce*” and “*Fichte*” will find “*oak*” and “*Eiche*” somewhere around. But it is highly unlikely that navigational and arboreal terms are organised similarly with respect to each other: since the relation between forestry and shipbuilding is relatively tentative in recent history, there is no reason to expect that those relations will hold across sets of training data. Other recent experiments demonstrate the challenge of aligning models built with different implementations (Søgaard et al., 2018) which to some extent should be expected to carry comparable information in spite of different encodings. It is not likely that a transformation that takes purchase in global structure is able to align two models of this type in the general case.

## Intrinsic Dimensionality

In whatever high-dimensional space the items of interest are embedded, their local neighbourhoods may be of much lower *intrinsic dimensionality*. This intrinsic dimensionality has to do with the complexity of the data under study. A collection of one-dimensional objects such as sticks or pieces of string is an example. Each point on each stick or string is perfectly defined by its position on the stick or string, which can be described in one single dimension. It may be practical to store all the sticks and strings in a three-dimensional container, such as a box. Each point on each stick and string is then in a three-dimensional space and its position can be specified using a three-dimensional vector in the box space—but the interesting characteristic of the point is still only its position on the one-dimensional object. Moving from sticks and strings to words and linguistic items, their local dimensionality is the question to be studied. The dimensionality of the global space—the box—may need to be high to accommodate a large number of semantic neighbourhoods, even while the intrinsic dimensionalities of those neighbourhoods are orders of magnitude smaller than the global dimensionality. Locally, the neighbourhoods do not make use of all those dimensions. Exploring this further would be in keeping with the previous point of the preeminence of local neighbourhoods over global structure. There is no obvious answer to how many relations to other items would be necessary to define the local neighbourhood. To specify the position of *turquoise*, one needs to relate it to a number of other hues and tints, and to constructions where they are used, but not to dinosaur species, teleological notions, or modal auxiliary verbs and verb-chain constructions. To specify the position of a modal auxiliary verb, the relations it enters into involve many constructions and a large variety of main verbs in very different ways than a colour term. One experiment, where 100,000 points in a semantic space were randomly chosen to have their local neighbourhood probed, verified the discrepancy between global and local dimensionality, and hypothesises local structures that are not apparent on a global level (Karlgren et al., 2008). In the case of linguistic items, the dimensionality of the box has been experimented with, not through a principled approach but through evaluation on linguistic tasks. If the global dimensionality is too low the compression of the data causes a language model to be noisy. This has been established variously depending on details of the implementational framework [on the order of a hundred, in the case of Latent Semantic Indexing (Deerwester et al., 1990), a few hundred for most current neural models, and on the order of a thousand for Sparse Distributed Memory (Kanerva et al., 2000)]. But the dimensionality of these “sticks” and “strings” has not been studied more closely: it is to be expected that some of the items have more convoluted local neighbourhoods than others.

## “When the Map Is More Exact Than the Terrain”

The geometrical toolkit is arbitrarily and deceptively precise even when the data may be fuzzy. As argued above, most pairs of items in a semantic space should be completely unrelated. If an other item is below the horizon of interest as viewed from one of interest, no answer should be given, rather than a precise float, as in Example (4).

4) What is the relation between *dodecahedron* and *chilly*?

Also, comparisons between distances may be meaningless, even when the model responds with exact scores. Examples (5-a) and (5-b) are questions that probably should not be answered, in contrast with Examples (2-a) and (2-b). When we use exact computations on approximate data, we risk believing the numerical results more than they deserve: the map is more exact than the terrain, as it were. This, of course, is true for any model that does precise computation on approximate data.

5) a. Is a *cow* more like a *donkey* than a *skillet* is like a *kettle*?
b. Is *Debussy* more like *Poulenc* than *Hank* is like *Waylon*?

A geometric model should be consulted with an awareness of the *semantic horizon* beyond which any relation is random noise: aggregating many such irrelevant signals between sets of items risks assuming a relation where none exists. A geometric model will deliver precise responses, but that precision is not always interpretable. Most importantly, a precise distance is not a measure of certainty. Neither is finding a low distance score between two items a measure of how confidently the model can assess their relation.

## When and How to Use Pre-Trained Embeddings

All the above admonitions apply equally to using pre-trained embeddings in a project. Frequently word vectors from other projects are used as input for experiments in e.g., text classification or topical analysis of material. This is in general a good idea. It allows an experiment to transcend the reliance on strings and to generalise from them to a somewhat more abstract level of meaning by using data from a larger body of language encoded in the vectors, by the reasoning given in *The Promise of Continuous Representations*. However, to do this, the experiment must make explicit what the hypothesis behind this generalisation step is, and then select (or retrain) the semantic vectors used accordingly. Is the objective to generalise from words to their synonym sets? Or is the objective to incorporate topical associations or connotations? These are all useful generalisations from a single word to a broader notion of linguistic meaning, but are not substitutable. Are the vectors trained on the same or at least comparable genre? Have the linguistic items been weighted by occurrence frequency or discriminatory power? A research project that includes a pre-trained semantic model should be able to state what aspect of meaning the model is intended to enhance.

## High-Dimensional Vector Spaces are Here to Stay

Processing of linguistic data in high-dimensional vector spaces has a history of over 40 years, and the concurrent increase in computing capacity and an accessible data has afforded impressive results in recent years. Besides the import of *geometry* to our understanding of semantics, which is the topic of this paper, the mathematics of high-dimensional spaces includes powerful computational *algebras* which allow for hypothesis-driven experimentation on semantic data. Such algebras are distinct from linear algebra and have simple operations for representing compositional structure (Plate, 2003) which allow combining semantics and syntax in a common computing architecture (Gayler, 2003) as shown in experiments that combine geometric and algebraic properties of high-dimensional representation (Jones and Mewhort, 2007; Joshi et al., 2017, e.g.). Statistical learning and rule-based inference are hallmarks of human intellectual information processing and a representation to handle both simultaneously is both possible and desirable.

## Footnotes

1 A similar point is made by Rekabsaz et al. (2017).
